# 

**Published:** 1868-02

**Authors:** 


### NOTICE.

The Mad River Valley Dental Association, will hold its next Quarterly Meeting in the city of Hamilton, Butler County, Ohio, the 1st Tuesday of April (being the 7th day) 1868, at the Hamilton House.

The following Essayists and subjects for discussion were appointed at our meeting:

### ESSAYISTS.

Dr. C. H. Harroun, Toledo; Dr. R. Corson, Middletown; J. H. Paine, Middletown; Dr. D. Phillips, Springfield.

### SUBJECTS FOR DISCUSSION.

1st. Anæsthesia, opened by Dr. Taft; 2d. Preparation of cavities for filling Teeth, opened by Dr. A. A. Blount; 3d. Miscellaneous business.

The following Resolution was passed and ordered to be inserted in the circulars next to be issued:

"That any active member who shall be absent from the Society twice in succession, and does not render a valid excuse or satisfactory explanation therefor, shall be expelled; for a like offence any officer may be superceded. Furthermore it shall be the duty of any member who cannot be present to send the Society a statement of the cause of his absence."

The election of officers will take place at the next meeting.

Dr. N. W. Williams, *President.*

J. H. Paine, *Corresponding Secretary.*
March 10th, 1868.

### OF THE WEST.

*Issued Monthly, at $3 a Year in Advance.*

DEVOTED TO THE INTERESTS OF THE DENTAL PROFESSION.

EDITED BY J. TAFT AND G. WATT.

The volume commences in January and closes in December.

The Register being issued monthly is always fresh, therefore indispensable to the practitioner who desires to keep posted up with the new inventions and improvements which are daily developed. Neither expense nor effort will be spared to give value and variety to its contents. Articles will be illustrated with well executed engravings whenever they will add to the interest or assist in explanation.

Its extensive circulation and frequency of issue makes it one of the BEST DENTAL ADVERTISING MEDIUMS in the United States.

RATES OF ADVERTISING.

One page, 1 year, $50. Half page, 1 year, $30. Quarter page, or card, 1 year $20.

All advertising bills payable in advance, or at furthest after the issue of the first number containing the advertisement. All transient advertisements to be paid for in advance.

☞ CONTRIBUTIONS SOLICITED.

Address, J. TAFT, or "DENTAL REGISTER," Cincinnati Ohio.

Business Communications to

J. TAFT, Publisher,
No. 238 Fourth St., between Plum and Central Avenue

